# Circulating LPS and (1→3)-β-D-Glucan: A Folie à Deux Contributing to HIV-Associated Immune Activation

**DOI:** 10.3389/fimmu.2019.00465

**Published:** 2019-03-18

**Authors:** Rayoun Ramendra, Stéphane Isnard, Vikram Mehraj, Jun Chen, Yonglong Zhang, Malcolm Finkelman, Jean-Pierre Routy

**Affiliations:** ^1^Chronic Viral Illness Service, McGill University Health Centre, Montreal, QC, Canada; ^2^Infectious Diseases and Immunity in Global Health Program, Research Institute, McGill University Health Centre, Montreal, QC, Canada; ^3^Department of Microbiology and Immunology, McGill University, Montreal, QC, Canada; ^4^Centre de Recherche du Centre Hospitalier de l'Université de Montréal, Montreal, QC, Canada; ^5^Associates of Cape Cod Inc., Falmouth, MA, United States; ^6^Division of Hematology, McGill University Health Centre, Montreal, QC, Canada

**Keywords:** HIV, LPS, (1→3)-β-D-Glucan, immune activation, microbial translocation, antiretroviral therapy, non-AIDS events

## Abstract

Immune activation is the driving force behind the occurrence of AIDS and non-AIDS events, and is only partially reduced by antiretroviral therapy (ART). Soon after HIV infection, intestinal CD4+ T cells are depleted leading to epithelial gut damage and subsequent translocation of microbes and/or their products into systemic circulation. Bacteria and fungi are the two most abundant populations of the gut microbiome. Circulating lipopolysaccharide (LPS) and (1→3)-β-D-Glucan (βDG), major components of bacterial and fungal cell walls respectively, are measured as markers of microbial translocation in the context of compromised gut barriers. While LPS is a well-known inducer of innate immune activation, βDG is emerging as a significant source of monocyte and NK cell activation that contributes to immune dysfunction. Herein, we critically evaluated recent literature to untangle the respective roles of LPS and βDG in HIV-associated immune dysfunction. Furthermore, we appraised the relevance of LPS and βDG as biomarkers of disease progression and immune activation on ART. Understanding the consequences of elevated LPS and βDG on immune activation will provide insight into novel therapeutic strategies against the occurrence of AIDS and non-AIDS events.

## Introduction

The gastrointestinal tract (GI) is a dynamic setting constantly in contact with nutrients, allergens, commensal microbes, and pathogens. As such, this milieu is equipped with a complex and well balanced system of physical and immunological barriers to allow the absorption of nutrients while preventing the translocation of microbes and their products (1). A physical barrier is formed by firmly linked intestinal epithelial cells (enterocytes) connected via tight junctions. These cells form villi to maximize the absorption of nutrients. The base of each villus forms crypts composed of intestinal stem cells and Paneth cells which secrete growth factors that promote intestinal stem cell proliferation, antimicrobial peptide secretion, and digestive enzyme production (2, 3). In the upper regions of the villus, goblet cells contribute to the physical and chemical barriers by secreting a mucous layer that protects the gut epithelium from the microbiota (4). Patrolling leukocytes in the lamina propria constitute an immunological barrier that ensures any pathogens in the lamina propria are phagocytosed, cleared, and sent to the draining mesenteric lymph nodes (5).

HIV-infection is characterized by the depletion of gut CD4+ T cells, epithelial gut damage, and translocation of microbes and their products into systemic circulation (6). People living with HIV (PLWH) have damage to the gut epithelium which has been shown to precede immune activation in models of SIV-infected rhesus macaques (7, 8). As systemic immune activation is considered the driving force of CD4+ T cell depletion and development of acquired immunodeficiency syndrome (AIDS), it is important to understand the link between epithelial gut damage and systemic immune activation in PLWH.

In 2006, Brenchley et al. were the first to report that increased plasma levels of gram-negative bacterial cell wall antigen lipopolysaccharide (LPS) induces systemic immune activation in both PLWH and SIV-infected rhesus macaques (9). Estes et al. in 2010, demonstrated that elevated plasma levels of LPS in PLWH is a result of HIV-induced epithelial gut damage allowing for the translocation of microbial products from the gut microbiota into systemic circulation (10). Despite the success of antiretroviral therapy (ART), epithelial gut damage, microbial translocation, and to a lesser extent systemic immune activation are not reversed. In parallel, Cani et al. coined the term “metabolic endotoxemia” to describe the phenomenon of obese individuals with high plasma levels of LPS linked to reduced insulin sensitivity and increased risk of metabolic diseases (11). Furthermore, conserved parts of the LPS molecule act as a pathogen-associated molecular patterns (PAMPs) that have been associated *in vitro*, in animal models, and epidemiologically with increased innate immune activation, inflammation, and risk of developing non-AIDS events in ART-treated PLWH (9, 12).

**Figure 1:**
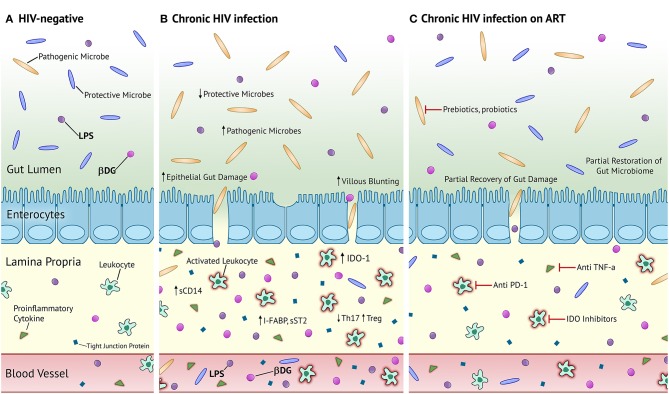
Proposed mechanism of microbial translocation in chronic HIV-infection and chronic HIV-infection on ART compared to HIV- homeostasis. **(A)** Healthy, HIV-uninfected individuals have an abundance of protective microbes in the gut and tight junctions connecting enterocytes to prevent the translocation of gut microbes and their products. Patrolling leukocytes in the lamina propria are not activated and there is an almost negligible level of inflammation and epithelial damage. **(B)** PLWH in the chronic stage of infection have increased proportions of pathogenic microbes and decreased proportions of protective microbes in the gut. There is severe villous blunting, epithelial gut damage, and microbial translocation. Patrolling leukocytes are activated and secrete large quantities of pro-inflammatory cytokines. There is elevated circulation of markers of epithelial gut damage, microbial translocation, and inflammation. **(C)** PLWH on long-term ART have partially restored composition of the gut microbiota, epithelial gut damage, and reduced microbial translocation. Activated leukocytes and systemic inflammation persists. Prebiotics and probiotics are potential therapeutic strategies to fully recover the composition of the gut microbiota. Anti PD-1, anti TNF-α, and IDO inhibitors represent potential therapeutic strategies against persistent immune activation and inflammation. LPS, lipopolysaccharide; βDG, (1→3)-β-D-Glucan; sCD14, soluble cluster of differentiation 14; I-FABP, intestinal fatty acid binding protein; sST2, soluble suppressor of tumorigenicity 2; IDO-1, indoleamine-2,3-dioxygenase 1.

Increasing awareness about the human gut microbiota reveals that it is a complex community of bacteria, fungi, archaea, viruses, and parasites influencing health and disease (13). However, studies regarding microbial translocation in PLWH have primarily focused on bacterial translocation. (1→3)-β-D-Glucan (βDG), a major component of most fungal cell walls, is commonly used as a biomarker for the diagnosis and management of invasive fungal infections (IFI) and has been recently used as a marker of fungal translocation in people without IFI (14). In 2012, Morris et al. were the first to show elevated plasma levels of βDG in PLWH (15). We and others have found that plasma levels of βDG are associated with epithelial gut damage, immune activation, inflammation, and risk of developing non-AIDS events (16–20). Like epithelial gut damage, plasma levels of βDG do not normalize despite long-term ART (20). These findings show converging evidence that like LPS, βDG also plays a significant role in chronic immune activation and development of non-AIDS events in PLWH (Figure 1).

Currently, persistence of systemic immune activation and development of non-AIDS events despite long-term ART represents one of the hurdles in caring for PLWH (21). Herein, we look to comprehensively review the existing English literature regarding the contribution of circulating LPS and βDG in systemic immune activation in PLWH. Understanding the consequences of LPS and βDG antigenemia will help with the development of therapeutic strategies against this “folie à deux.”

## Gut Damage

In homeostatic conditions, the microbiota is contained within the gut by the mucous layer, epithelial barrier, and residential leukocytes (22–25).

HIV infection leads to early disruption of the gut epithelial barrier characterized by villous atrophy, crypt hyperplasia, increased gastrointestinal inflammation, and increased intestinal permeability (7, 26–28). Such damage is not completely restored despite long-term ART. Deterioration of the gastrointestinal landscape in PLWH and SIV-infected rhesus macaques has been shown to cause microbial translocation and resultant immune activation (9, 10, 29). Interestingly, SIV-infected sooty mangabeys, which do not progress to AIDS, present without disruption of the GI epithelial layer nor increased microbial translocation, and limited immune activation (30, 31). Thus, understanding the cause(s) and implications of epithelial gut damage in PLWH may help to understand the source(s) of systemic immune activation.

The precise mechanisms responsible for HIV-associated epithelial gut damage remain poorly understood and are now known to precede mucosal immune dysfunction (7). HIV gp120 and Tat proteins have been shown to have detrimental effects on intestinal epithelial cells (32–36). HIV induces inflammasome to produce IL-18, resulting in intestinal epithelial cell death and reduced expression of tight junction proteins, contributing to intestinal permeability and resultant microbial translocation (37, 38). Globally, HIV contributes to epithelial gut damage which is partially improved on ART (39, 40). Markers of epithelial gut damage such as soluble suppressor of tumorigenicity 2 (sST2) and intestinal fatty acid binding protein (I-FABP) have been reported to be elevated in inflammatory bowel diseases, graft vs. host disease, and HIV (41, 42).

As reported by Hensley-McBain et al., alterations to the intestinal epithelial structure precede mucosal immune dysfunction (7). Early mucosal damage in PLWH is partially explained by a substantially high expression of CCR5, a HIV co-receptor, on CD4+ T cells in the gut as compared to peripheral blood (43, 44). This is in line with findings that HIV is 10 times more likely to infect CD4+ T cells in the gut compared to peripheral blood (45). IL-17 producing cells, such as Th17 and Th22 CD4+ T cells, are known to homeostatically maintain the epithelial barrier (7). We and others have reported alterations of the Th17/Treg ratio in PLWH, owing to increased frequency of Tregs and decreased frequency of Th17 CD4+ T cells (46, 47). This in turn impairs the homeostatic response to prevent and restore epithelial gut damage in PLWH. HIV-associated gastrointestinal abnormalities have also been associated with changes in the composition of the gut microbiome (dysbiosis) and translocation of microbial products in PLWH.

## Bacterial and Fungal Translocation

In homeostatic conditions, microbial translocation is limited by physical and immunological barriers in the intestine (48). However, when the gut epithelium is damaged and the mucosal immune system is compromised, microbial products translocate out of the gut into systemic circulation via the lamina propria (49, 50). Microbial translocation has been implicated in several conditions including Crohn's disease, ulcerative colitis, graft-vs.-host disease, and HIV (40).

SIV-infected rhesus macaques were shown to have elevated plasma levels of LPS that could be partially reversed with antibiotics. Immunohistochemistry and immunofluorescence of gut biopsies demonstrated that such elevation was a result of bacterial translocation from the gut lumen into systemic circulation via the lamina propria (9, 10). Interestingly, SIV-infected African green monkeys, a natural host of SIV, receiving intravenous injection of LPS had increased viral replication, mucosal CD4+ T cell depletion, and systemic immune activation without inducing epithelial gut damage (51, 52). Altogether, this suggests that translocation of microbial products plays a major role in systemic immune activation and inflammation.

We and others have shown elevation of microbial products in the blood of PLWH (9, 15, 46). Most published studies measured microbial translocation using plasma levels of bacterial DNA fragments, LPS, LPS binding protein (LBP), soluble CD14 (sCD14), and EndoCab (50). Bacterial DNA fragments were quantified using 16S rDNA PCR. Most studies in PLWH use the Limulus Amebocyte Lysate (LAL) assay to measure plasma levels of LPS, a major component of the outer membrane of gram-negative bacteria. LPS binds to LBP, which transfers LPS onto monocytes/macrophages causing the release of soluble CD14 (sCD14) (53). EndoCab is a group of antibodies specific for the core of LPS that are produced by B cells in response to enteric gram-negative bacteria (54). LBP, sCD14, and EndoCab are commonly measured in plasma/serum using solid-phase enzyme-linked immunosorbent assay (ELISA) as circulating biomarkers of LPS-induced innate immune activation (9, 39).

Most research on microbial translocation in PLWH has been focused on bacterial translocation (13). Morris et al. were the first to show elevation of circulating levels of fungal cell wall antigen, βDG, in PLWH (15). Hoenigl et al. have shown that plasma βDG is inversely correlated with the abundance of *Lactobacilli* in the distal gut (19). *Lactobacillus* is a protective genus of bacteria that is inversely associated with gut integrity and distal gut permeability. Furthermore, we have shown that plasma levels of βDG is strongly correlated with classical marker of epithelial gut damage, I-FABP, and markers of microbial translocation LPS and sCD14 (20) (Table 1). The origin of circulating βDG was first studied in murine models. As opposed to the human gut microbiome, there is no *Candida* in the murine gut. Mice fed with live or heat-inactivated *Candida* had elevated serum levels of βDG. Such elevation induced the production of pro-inflammatory cytokines IL-6 and TNF-α. Administration of Fluconazole, an anti-fungal small molecule, partially inhibited the elevation of serum βDG and systemic inflammation (55). Similar results were found in a murine model of lupus and sepsis (56, 57). As PLWH without invasive fungal infections are highly susceptible to increased proportions of fungal colonization and have high levels of epithelial gut damage, it is highly likely that elevated plasma levels of βDG in PLWH originates from the gut (14). Thus, there is a need to definitely determine whether elevated plasma levels of βDG in PLWH is a result of microbial translocation and involved in systemic immune activation.

**Table 1:** Overview of studies associating elevation of plasma levels of βDG with immune activation and immune dysfunction in PLWH.

**References (country)**	**Sample size**	**Study populations**	**Major findings**
Morris et al. (15) (USA)	132	Chronic ART-treated PLWH; cross-sectional analysis	βDG was elevated in the plasma of PLWH and associated with plasma levels of IL-8, TNF-α, and frequency of CD38+ and HLA-DR+ CD8+ T cells. Elevated βDG was associated with cardiopulmonary dysfunction.
Hoenigl et al. (17) (USA)	41	Chronic ART-treated PLWH; cross-sectional analysis	Plasma level of βDG was positively associated with plasma levels of neopterin and IL-6.
Hoenigl et al. (19) (USA)	11	PLWH in early stage of infection, before and after ART; cross-sectional analysis	Elevated plasma levels of βDG was inversely correlated with abundance of *Lactobacillales* in the distal gut.
Hoenigl et al. (16) (USA)	21	Chronic ART-treated PLWH; cross-sectional analysis	βDG was elevated in the plasma and CSF of PLWH and positively associated with neurocognitive dysfunction.
Hoenigl et al. (18) (USA)	451	PLWH before and after ART; cross-sectional analysis	Multivariate analysis showed that pre-event plasma levels of βDG and LBP was independently associated with increased risk of non-AIDS events.
Mehraj et al. (20) (Canada)	146	PLWH in early and chronic stages, ART-treated and untreated; longitudinal and cross sectional analysis	Plasma levels of βDG was associated with plasma viral load, I-FABP, LPS, markers of IDO-1 metabolism, and frequency of Tregs. Expression of βDG-specific receptors, Dectin-1 and NKp30, was inversely correlated with plasma levels of βDG but not LPS. PLWH who initiated ART early had lower levels of plasma βDG and elevated βDG did not normalize despite long-term ART.

*PLWH, People living with HIV; ART, Antiretroviral Therapy; βDG, (1→3)-β-D-Glucan; LPS, Lipopolysaccharide; LBP, LPS Binding Protein; I-FABP, Intestinal Fatty Acid Binding Protein (marker of epithelial gut damage)*.

## Microbial Translocation and Systemic Immune Activation

Microbial products such as LPS and βDG represent potent PAMPs that trigger a significant immune response. Several studies have provided convincing evidence that elevated plasma levels of LPS induces immune activation in sepsis, Crohn's disease, ulcerative colitis, obesity, and HIV (9, 11, 58, 59).

LPS is captured by LBP and complexed with CD14, myeloid differentiation 2 protein, and Toll like receptor 4 (TLR4). The formation of this complex is crucial for the immune system to mount a response to LPS. Classical antigen presenting cells (APCs) recognize this complex using TLR4 and subsequently phagocytose LPS while shedding sCD14. B cells also express TLR4 to recognize different parts of the LPS core lipids and secrete EndoCAb to facilitate the phagocytosis of the antigen (60). LPS has been shown to induce the secretion of several pro-inflammatory cytokines including IL-6, IL-8, and TNF-α by APCs (61). Plasma levels of LPS correlated with plasma levels of IFN-α and frequency of activated CD4+ and CD8+ T cells in PLWH (9). Similarly, plasma levels of sCD14 correlated with plasma levels of IL-6 and C reactive protein in PLWH (62).

Meanwhile, βDG is predominantly recognized by complement receptor 3 (CR3), Dectin-1, NKp30, Ephrin type-A receptor 2 (EphA2), and Langerin. CR3 is a ubiquitous heterodimer receptor composed of CD11b and CD18. Recent findings have shown reduced expression of CR3 on both myeloid and plasmacytoid dendritic cells (DC) in PLWH. βDG-specific interactions with CR3 on DC have been shown to increase IL-6 and TNF-α production by activating the Syk-JNK-AP-1 pathway (63). Dectin-1 represents the most prominent myeloid cell receptor for βDG and is expressed on monocytes, macrophages, DCs, and neutrophils (63–65). We have shown that Dectin-1 expression on monocytes is reduced in PLWH and that such expression is inversely correlated with plasma levels of βDG but not LPS (20). βDG-specific binding to Dectin-1 leads to the production of pro-inflammatory cytokines IL-6, IL-8, and TNF-α by myeloid cells (66, 67). βDG is also specifically recognized by NK cells via NKp30, a functional activation receptor (68). In addition, we have shown that NKp30 expression is diminished in PLWH and inversely correlated with plasma levels of βDG but not LPS (20, 69). NKp30-specific binding has been shown to induce activation and the production of pro-inflammatory cytokines such as IL-1β and TNF-α (70, 71). EphA2 is a βDG-specific receptor expressed on epithelial cells, predominantly in the colon and small intestine, that has yet to be measured in PLWH (72). Interestingly, EphA2 has also been identified as a receptor for Kaposi Sarcoma associated herpes virus (also called HHV8), one of the most common HIV-associated co-infections (73). DCs play an important role in maintaining mucosal homeostasis. In the mucosa, they can be distinguished according to their expression of C-type lectins: Langerin [expressed by Langerhans cells (LCs)] and DC-SIGN (expressed by classical DCs). LCs reside in the epithelium of most mucosal surfaces and are thus one of the first APCs to encounter HIV as well as products of microbial translocation. Langerin has been shown to be an important receptor for βDG during *Candida* and *Saccharomyces* infections that has yet to be assessed in PLWH without IFI (74). βDG induces the secretion of pro-inflammatory cytokines IL-1β, IL-6, IL-8, IL-23, TNF-α, and chemokine CCL22 that has been shown to increase monocyte recruitment to the colon (66, 75, 76). Indeed, we and others have shown that elevated plasma levels of βDG is correlated with plasma levels of IL-6 and IL-8 in PLWH (15, 20).

Microbial translocation in PLWH is associated with Indoleamine-2,3-deoxygenase-1 (IDO-1) activity and HIV disease progression (77). IDO-1 is expressed in all myeloid cells and activated after PAMPs recognition to metabolize Tryptophan into Kynurenines (78). As such, IDO-1 activity is considered a marker of inflammation and immune activation. We and others have shown that IDO-1 activity is increased in PLWH and does not normalize with early ART. In PLWH, IDO-1 activity is associated with plasma levels of LPS and βDG, increased frequency of Tregs, epithelial gut damage, microbial translocation, immune activation, and HIV reservoir size (20, 46, 79, 80).

Persistent epithelial gut damage and elevated plasma levels of LPS and βDG, despite long-term ART, likely contribute to inflammation and chronic immune activation leading to the development of non-AIDS events in PLWH. In the ART-era, the development of non-AIDS events represents one of the challenges to caring for PLWH. Therefore, both LPS and βDG represent important therapeutic targets to reduce the risk of developing non-AIDS events.

## Microbial Translocation, Inflammation, and non-AIDS Events

Despite the significant success of ART, PLWH still present with high rates of non-AIDS events that includes HIV-associated neurocognitive disorders (HANDs), cardiovascular diseases, renal failure, liver steatosis, and cancer (81, 82). Such non-AIDS events have been associated with epithelial gut damage, microbial translocation, and systemic immune activation (50). Hoenigl et al. have observed in a large cohort of ART-treated PLWH that in addition to soluble urokinase-type plasminogen receptor, plasma levels of βDG, and LBP represent two of the best predictors of increased risk of non-AIDS events (18).

### Microbial Translocation and HAND

PLWH present with HANDs including asymptomatic neurocognitive impairment, mild neurocognitive disorder, and dementia (83, 84). Previous studies have found strong associations between plasma levels of sCD14, LPS, and βDG with neurocognitive dysfunction (16, 85). Moreover, ART-treated PLWH with severe neurocognitive dysfunction also presented with elevated sCD14 and βDG in their cerebrospinal fluid (CSF) (16). Supporting the concept of the gut-brain axis, increased microbial translocation likely plays a crucial role in the development of HANDs.

### Microbial Translocation and Cardiovascular Diseases

A study with more than 27,000 participants showed that PLWH had a two-fold increased risk of developing acute myocardial infarction in every age group compared to matched control participants (86, 87). Elevated circulation of microbial products and resultant inflammation are associated with increased risk of heart disease (88). Plasma levels of LPS have been associated with known risk factors for cardiovascular diseases such as decreased insulin sensitivity and higher total cholesterol (11). Similarly, elevated plasma levels of βDG have also been associated with cardiopulmonary dysfunction (15).

## Conclusions and Future Directions

Due to the success of ART, the life expectancy and quality of life of PLWH has dramatically improved over the course of the last decade. While early initiation of ART is associated with lower reservoir size and reduced immune activation, PLWH on ART still suffer from unrecovered epithelial gut damage and chronic immune activation (89). It has been recently reported that epithelial gut damage precedes systemic immune activation in a SIV-infected rhesus macaque model (7). Like epithelial gut damage and systemic immune activation, markers of microbial translocation do not normalize despite long-term ART (20). Thus, understanding the mechanisms via which epithelial gut damage and resultant microbial translocation contribute to chronic immune activation is essential toward improving the prognosis of PLWH.

Circulating microbial polysaccharides LPS and βDG are elevated in PLWH (9, 15). Previous research on microbial translocation in PLWH and SIV-infected rhesus macaques has been primarily measured by plasma levels of LPS using the LAL assay. Of note, it has been initially found that LPS measured by the LAL assay also measures βDG (90). While it has been shown that increased plasma levels of LPS are a result of microbial translocation in PLWH, the source of circulating βDG remains to be clarified. Consumption of certain mushrooms, oat fiber, and seaweed can also increase circulating levels of βDG (91). We and others have shown that plasma levels of βDG are positively associated with marker of epithelial gut damage I-FABP and inversely associated with the abundance of protective bacteria, *Lactobacillales*, in the distal gut (19, 20). Furthermore, murine models have shown that mice fed with heat-killed or live *Candida* have increased levels of circulating βDG after cecal ligation and puncture (55). Similarly, people with intestine disorders have increased plasma levels of βDG (56, 92, 93). Hence, there is converging evidence suggesting that elevated plasma levels of βDG in PLWH are a result of increased *Candida* or other fungal colonization, epithelial gut damage, and subsequent microbial translocation.

Both LPS and βDG have been associated with markers of systemic immune activation, inflammation, and the development of non-AIDS events. As such, the individual and potentially synergistic consequences of elevated plasma levels of LPS and βDG on systemic immune activation must be urgently addressed.

Understanding the respective mechanisms by which these two microbial polysaccharides contribute to chronic immune activation may lead to the development of novel therapeutic strategies against inflammation and the development of non-AIDS events in PLWH. Moreover, genetic factors may play a key role in determining the influence of these PAMPs on systemic immune activation. For example, people with CARD9 deficiencies have been shown to have increased susceptibility to fungal infections (94). Furthermore, Palesch et al. demonstrated that sooty mangabeys, a natural host of SIV, had a frameshift mutation in their TLR4 gene that was associated with a blunted response to TLR4 ligands *in vitro* (95). Thus, regular genetic variations in receptors for LPS and βDG may also play a pivotal role in determining the consequences of microbial translocation in PLWH.

In 2014, Kristoff et al. gave Sevelamer, a drug known to decrease circulating LPS (96), to SIV-infected pigtailed macaques. A single dose administration led to partially decreased HIV viral replication, decreased circulation of coagulation markers, and decreased immune activation/inflammation (52). However, when Sandler et al. gave Sevelamer to 36 ART-naïve PLWH, they did not observe a decrease in plasma levels of LPS (measured by LAL assay) nor markers of immune activation/inflammation (97). Future clinical trials should aim to reduce the burden of elevated circulation of products of both bacterial and fungal translocation. While therapeutic strategies targeting elevated circulation of PAMPs should be investigated, future studies should also look to target immune signaling molecules (such as TLR4 and Dectin-1) to reduce LPS and βDG induced systemic immune activation.

Overall, elevated plasma levels of LPS and βDG both play an important role in chronic immune activation in PLWH on long-term ART and may represent a “folie à deux” contributing to the development of non-AIDS events. To this end, gaining a comprehensive understanding of the origin and consequences of these circulating microbial polysaccharides is of critical importance to finding therapeutic strategies to restore mucosal homeostasis, and gut dysbiosis in PLWH.

## Author Contributions

RR made the first draft, constructed the figure and table, and made revisions to the final draft of the manuscript. RR and SI contributed significantly to conducting the literature review. VM, JC, YZ, and MF critically read and revised the manuscript. J-PR designed the review and critically revised the manuscript.

### Conflict of Interest Statement

MF and YZ are employees of Associates of Cape Cod, Inc., the manufacturers of Fungitell, the (1→3)-β-D-Glucan *in vitro* diagnostic kit. The remaining authors declare that the research was conducted in the absence of any commercial or financial relationships that could be construed as a potential conflict of interest.
